# Beneficial effect of aurothiomalate on murine malaria

**DOI:** 10.1186/1475-2875-9-118

**Published:** 2010-05-07

**Authors:** Ioana Alesutan, Diwakar Bobbala, Syed M Qadri, Adriana Estremera, Michael Föller, Florian Lang

**Affiliations:** 1 Department of Physiology, University of Tübingen, Gmelinstr. 5, 72076 Tübingen, Germany

## Abstract

**Background:**

Premature death of *Plasmodium*-infected erythrocytes is considered to favourably influence the clinical course of malaria. Aurothiomalate has previously been shown to trigger erythrocyte death or eryptosis, which is characterized by cell membrane scrambling leading to phosphatidylserine exposure at the cell surface. Phosphatidylserine-exposing cells are rapidly cleared from circulating blood. The present study thus tested whether sodium aurothiomalate influences the intraerythrocytic parasite development *in vitro *and the clinical course of murine malaria *in vivo*.

**Methods:**

Human erythrocytes were infected with *Plasmodium falciparum *BinH *in vitro *and mice were infected (intraperitoneal injection of 1 × 10^6 ^parasitized murine erythrocytes) with *Plasmodium berghei *ANKA *in vivo*.

**Results:**

Exposure to aurothiomalate significantly decreased the *in vitro *parasitemia of *P. falciparum*-infected human erythrocytes without influencing the intraerythrocytic DNA/RNA content. Administration of sodium aurothiomalate *in vivo *(daily 10 mg/kg b.w. s.c. from the 8^th ^day of infection) enhanced the percentage of phosphatidylserine-exposing infected and noninfected erythrocytes in blood. All nontreated mice died within 30 days of infection. Aurothiomalate-treatment delayed the lethal course of malaria leading to survival of more than 50% of the mice 30 days after infection.

**Conclusions:**

Sodium aurothiomalate influences the survival of *Plasmodium berghei*-infected mice, an effect only partially explained by stimulation of eryptosis.

## Background

The malaria pathogen *Plasmodium *imposes oxidative stress on infected cells [1], which in turn elicits eryptosis, a form of erythrocyte death [2-4]. The signaling leading to cell membrane scrambling includes an increase in the cytosolic Ca^2+ ^activity [5-10] and/or formation of ceramide and/or formation of ceramide [11]. Ca^2+ ^further stimulates Ca^2+^-sensitive K^+ ^channels [10,12-18]. The Ca^2+^-permeable cation channels are activated by oxidative stress [19,20]. Oxidative stress [21] and excessive cytosolic Ca^2+ ^concentrations [22] are known to similarly trigger cell membrane scrambling or apoptosis in nucleated cells.

Phosphatidylserine-exposing cells are recognized by macrophages [23,24], phagocytosed [25,26] and thus rapidly cleared from circulating blood [27].

In malaria, accelerated clearance of infected erythrocytes [28] may counteract the development of parasitemia [29-31], in genetic erythrocyte disorders [9,32-36], in iron deficiency [2], or following treatment with lead [3], chlorpromazine [37], azathioprine [38] or cyclosporine [39]. The erythrocyte cation channel is inhibited by erythropoietin [40] and may favourably influence the course of malaria [15]. Eryptosis is further inhibited by erythropoietin [41], caffeine [42] and thymol [43].

Eryptosis is stimulated by aurothiomalate, a gold-containing drug effective against rheumatoid arthritis [44]. Gold complexes have indeed been shown to counteract malaria [45-51]. They are considered to be effective through inhibition of heme aggregation, haemozoin formation and/or parasitic thioredoxin reductase as well as interaction with the DNA of the parasite [52-57].

The present study explored, whether sodium aurothiomalate augments the death of *Plasmodium falciparum*-infected human erythrocytes and/or *Plasmodium berghei*-infected mouse erythrocytes and whether this effect correlates with a favourable influence on parasitemia and host survival during murine malaria.

## Methods

Human erythrocytes were drawn from healthy volunteers. The study was approved by the Ethical commission of the University of Tübingen.

Animal experiments were performed according to the German animal protection law and approved by the local authorities (registration number PY 4/09). Experiments were performed in healthy SV129/J wild type mice (aged 4 months, both male and female). The animals had free access to standard chow (C1310, Altromin, Lage, Germany) and drinking water. Blood was drawn by incision of the tail vein.

For infection of human erythrocytes the human pathogen *Plasmodium falciparum (P. falciparum) *strain BinH [58] was grown *in vitro *[37,59]. Parasites were cultured as described earlier [60-62] at a hematocrit of 2% and a parasitemia of 2-10% in RPMI 1640 medium supplemented with Albumax II (0.5%; Gibco, Karlsruhe, Germany) in an atmosphere of 90% N_2_, 5% CO_2 _and 5% O_2 _[62,63].

To estimate the *in vitro *growth of *Plasmodium falciparum *the BinH strain was cultured and synchronized to the ring stage by sorbitol treatment as described previously [14,63]. For the *in vitro *growth assay, synchronized parasitized erythrocytes were aliquoted in 96-well plates (200 μl aliquots, 1% hematocrit, 0.5-2% parasitemia) and grown for 48 h in the presence or absence of sodium aurothiomalate.

The parasitemia was assessed 0 h and 48 h after infection by flow cytometry of human erythrocytes and by counting of Giemsa-stained blood smears from infected mice. Parasitemia was defined as the percentage of erythrocytes stained with the DNA/RNA-specific fluorescence dye Syto16 or by identification of Giemsa-stained infected erythrocytes using light microscopy.

For Giemsa staining, the thick blood film was air-dried and fixed with methanol. 2% Giemsa solution (Sigma) was added for 30 min. The slide was rinsed with water and again dried. Then, the slides were analysed under a Leica CM E light microscope (100 ×, oil immersion).

To estimate DNA/RNA amplification of the intraerythrocytic parasite, the culture was ring stage-synchronized and re-synchronized after 6 h of culture (to narrow the developmental parasite stage), aliquoted (200 μl aliquots, 2% hematocrit and 10% parasitemia) and cultured for further 16 h in the presence or absence of sodium aurothiomalate. Thereafter, the DNA/RNA amount of the parasitized erythrocytes was determined by Syto16 fluorescence as a measure of intraerythrocytic parasite copies.

For infection of mice *Plasmodium berghei *ANKA-parasitized murine erythrocytes (1 × 10^6^) were injected intraperitoneally [64,65]. Where indicated, sodium aurothiomalate (10 mg/kg b.w. s.c) was administered from the 8^th ^day of infection daily. Blood was collected from the mice daily starting 8 days after infection by incision of the tail. The hematocrit was determined by centrifugation in hematocrit capillaries. Parasitemia was determined by Syto16 staining in FACS analysis. *In vitro *experiments were performed at 37°C in Ringer solution containing (in mM) 125 NaCl, 5 KCl, 1 MgSO_4_, 32 N-2-hydroxyethylpiperazine-N-2-ethanesulfonic acid (HEPES)/NaOH (pH 7.4), 5 glucose, 1 CaCl_2 _[66]. Aurothiomalate was added to the NaCl Ringer at final concentrations varying from 0.1 to 100 μM (Sigma, Schnelldorf, Germany). For *in vitro *treatment, the final hematocrit was adjusted to 0.3%.

For determination of phosphatidylserine exposure, FACS analysis was performed as described [10]. After incubation in the presence or absence of sodium aurothiomalate, suspensions of *Plasmodium falciparum-*infected erythrocytes were stained with annexin V-APC (BD Biosciences Pharmingen, Heidelberg, Germany) and/or with Syto16 (Molecular Probes, Göttingen, Germany) to identify phosphatidylserine-exposing and infected erythrocytes, respectively. For annexin V-binding, erythrocytes were washed, resuspended in annexin V-binding buffer (Ringer solution containing 5 mM CaCl_2_. pH 7.4), stained with annexin V-APC (dilution 1:20), incubated for 20 min at room temperature, and diluted 1:5 with annexin V-binding buffer. Syto16 (final concentration of 20 nM) was added directly to the diluted erythrocyte suspension or co-incubated in the annexin V-containing buffer solution. Erythrocytes were analyzed by flow cytometry (FACS-Calibur, Becton Dickinson, Heidelberg, Germany) in fluorescence channel FL-1 for Syto16 (detected at 530 nm) and in FL-4 for annexin V-APC fluorescence intensity (detected at 660 nm).

Data are expressed as arithmetic means ± SEM, and statistical analysis was made by t-test or ANOVA using Tukey's test as post hoc test, as appropriate. p < 0.05 was considered as statistically significant.

## Results and Discussion

**Figure 1:**
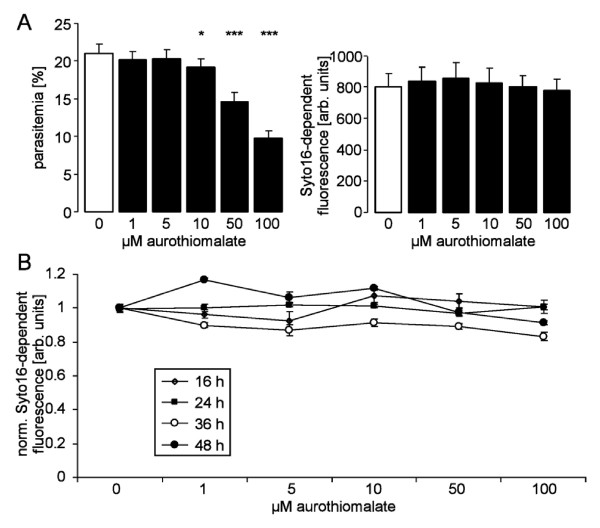
**Effects of sodium aurothiomalate on intraerythrocytic amplification and *in vitro *parasitemia**. **A**. *In vitro *parasitemia with *P. falciparum *(left panel) in human erythrocytes as a function of the aurothiomalate concentration (arithmetic means ± SEM, n = 16). *, *** indicate significant difference (p < 0.05, p < 0.001) from the absence of aurothiomalate. Intraerythrocytic DNA amplification (right panel) as a function of the aurothiomalate concentration (arithmetic means ± SEM, n = 12). **B**. Intraerythrocytic DNA amplification (right panel) as in B for different time periods (arithmetic means ± SEM, n = 8).

A first series of experiments explored the influence of aurothiomalate on the *in vitro *growth of *Plasmodium falciparum *in human erythrocytes. To this end, *P. falciparum*-infected erythrocytes were cultured in human erythrocytes and synchronized to the ring stage by sorbitol treatment. Within 48 hours the percentage of infected erythrocytes increased from 6.0% to 21.0% in the absence and to 9.8% in the presence of 100 μM aurothiomalate (Fig. 1A). Accordingly, aurothiomalate blunted the increase in the percentage of parasitized erythrocytes, an effect reaching statistical significance at ≥ 10 μM aurothiomalate (Fig. 1A). The halfmaximal inhibition (IC50) was achieved by 68 μM aurothiomalate. In contrast, at the concentrations tested, the presence of aurothiomalate did not influence the intraerythrocytic DNA amplification of the parasite (Fig. 1AB).

**Figure 2:**
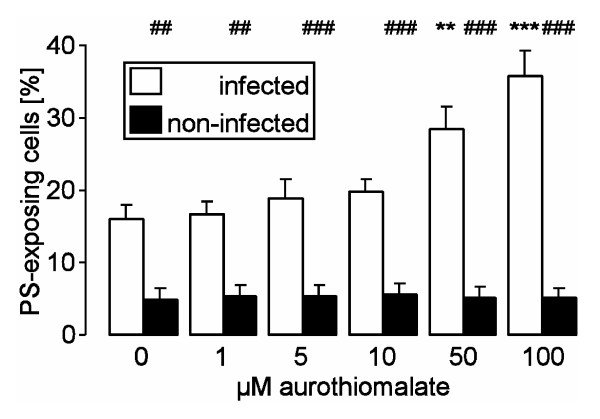
**Effects of aurothiomalate on phosphatidylserine exposure of infected and noninfected human erythrocytes**. Arithmetic means ± SEM (n = 6) of the percentage of annexin V-binding infected (open bars) and non-infected (closed bars) erythrocytes following infection of human erythrocytes with *P. falciparum *in the presence of 0 - 100 μM aurothiomalate. ##, ### indicate significant difference (p < 0.01, p < 0.001) from non-infected erythrocytes, **, *** indicate significant difference (p < 0.01, p < 0.001) from the absence of aurothiomalate.

In order to determine the effect of infection and of aurothiomalate on eryptosis, phosphatidylserine-exposing erythrocytes were identified by measurement of annexin V-binding in FACS analysis. Within 24 hours, the infection with *P. falciparum *markedly increased the annexin V-binding of infected and noninfected erythrocytes (Fig. 2). The percentage of annexin V-binding was, however, significantly higher in infected than in noninfected erythrocytes (Fig. 2). The phosphatidylserine exposure of infected erythrocytes was significantly increased in the presence of aurothiomalate (Fig. 2), an effect reaching statistical significance at 50 μM aurothiomalate.

**Figure 3:**
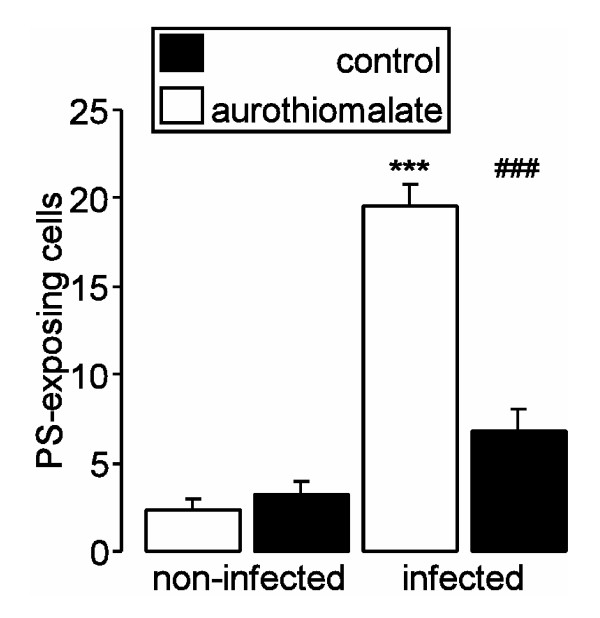
**Effect of sodium aurothiomalate treatment on phosphatidylserine exposure of infected and non-infected erythrocytes from *Plasmodium berghei*-infected mice**. Arithmetic means ± SEM (n = 6-8) of the percentage of phosphatidylserine-exposing infected (right bars) and non-infected (left bars) erythrocytes taken from animals without (black bars) and with (white bars) sodium aurothiomalate treatment (daily 10 mg/kg b.w. s.c.) on the 22^nd ^day after infection. ### indicates significant difference (p < 0.001) from absence of sodium aurothiomalate. *** indicates significant difference (p < 0.001) from noninfected erythrocytes.

In a next series, mice were infected with *P. berghei *with or without sodium aurothiomalate treatment. Sodium aurothiomalate was administered daily from the 8^th ^day of infection. Similar to the *in vitro *infection of human erythrocytes with *P. falciparum*, the infection of mice with *P. berghei *was followed by a marked increase in the percentage of phosphatidylserine-exposing erythrocytes. The phosphatidylserine exposure of infected erythrocytes was significantly more pronounced following treatment with sodium aurothiomalate than the phosphatidylserine exposure of noninfected erythrocytes (Fig. 3).

**Figure 4:**
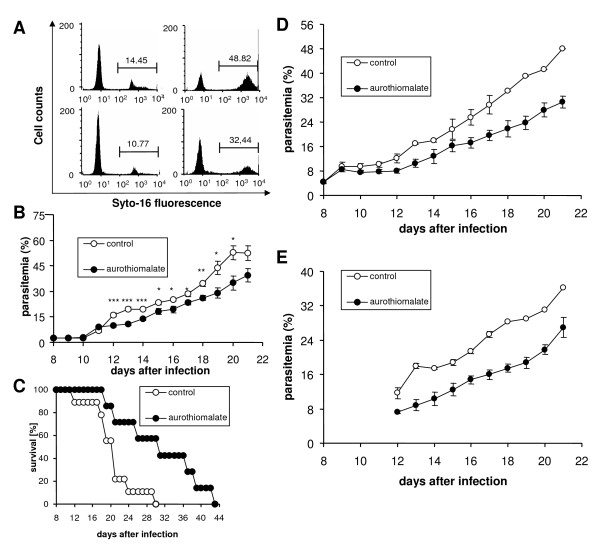
**Effect of sodium aurothiomalate treatment on the parasitemia and survival of *Plasmodium berghei*-infected mice**. **A: **Original histograms of parasitemia-dependent Syto16 fluorescence in untreated animals (upper panels) and in animals treated daily from day 8 daily with 10 mg/kg b.w. sodium aurothiomalate s.c. (lower panels) 10 (left panels) and 20 (right panels) days after infection with *P. berghei*. **B: **Arithmetic means ± SEM of the parasitemia in mice without treatment (open circles, n = 8 mice) or with daily 10 mg/kg b.w. s.c. of sodium aurothiomalate (closed circles, n = 6 mice) as a function of days after infection with *P. berghei*. Significant difference (* p < 0.05, ** p < 0.01, *** p < 0.001; t-test) from the untreated animals on days 12 - 20. The results presented are one of three independent series. **C: **Survival of mice without treatment (open circles) or with daily 10 mg/kg b.w. sodium aurothiomalate s.c. (closed circles) as a function of days after infection with *Plasmodium berghei*. **D-E**: Arithmetic means ± SEM of the parasitemia in mice without treatment (open circles, n = 4 mice) or with daily 10 mg/kg b.w. s.c. of sodium aurothiomalate (closed circles, n = 4 mice) as a function of days after infection with *P. berghei*. The parasitemia was determined daily either by staining with Syto16 and subsequent FACS analysis as in B (**D**) or by daily Giemsa staining of blood smears and light microscopy-dependent analysis (**E**).

The parasitemia was still low on the 8^th ^day of infection (Fig. 4B). The percentage of infected erythrocytes gradually increased with or without sodium aurothiomalate treatment. However, the percentage of parasitized erythrocytes was significantly lower in sodium aurothiomalate-treated animals than in animals without sodium aurothiomalate treatment (Fig. 4A, B).

Since the FACS-dependent determination of parasitemia utilizes a DNA/RNA-specific dye, reticulocytes may also be counted as parasitized erythrocytes. Therefore, a second series of experiments was performed to compare the values for parasitemia determined by FACS analysis to those obtained from Giemsa staining. As shown in Fig. 4D, E, parasitemia was lower in the aurothiomalate-treated group of mice, irrespective of the methods applied.

The treatment with sodium aurothiomalate further resulted in enhanced survival of *P. berghei*-infected mice. As shown in Fig. 4C, all untreated animals died within 30 days after the infection. In contrast, 57% of the sodium aurothiomalate-treated animals were still alive 30 days after infection. All treated mice died, however, until day 44 after infection.

To investigate whether aurothiomalate treatment influences inflammation, the plasma levels of the inflammatory mediator TNFα, were determined on the 16^th ^day of infection. As a result, the TNFα concentration in non-treated mice was 53.4 ± 29.7 pg/ml whereas the TNFα was below the detection limit in mice treated with sodium aurothiomalate (both n = 4).

As shown earlier, TNF-α may exert an antiparasitic effect in animal models [67-69], and high TNF production is associated with more rapid clinical and parasitologic recovery in humans [70]. Even though aurothiomalate does not seem to affect induction of TNF-alpha in phagocytic cell cultures [71], the present observations clearly demonstrate an effect of the drug on TNF production *in vivo*.

**Figure 5:**
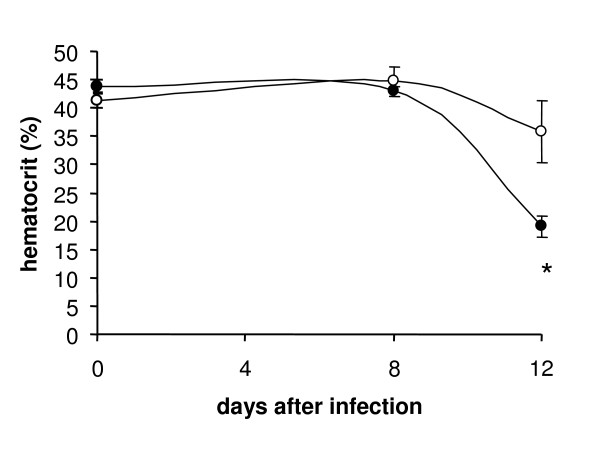
**Effect of sodium aurothiomalate treatment on the hematocrit of *Plasmodium berghei*-infected mice**. Arithmetic means ± SEM of packed cell volume (hematocrit) in mice without treatment (open circles, n = 8 mice) or with daily 10 mg/kg b.w. s.c. of sodium aurothiomalate (closed circles, n = 8 mice) as a function of days after infection with *P. berghei*. * indicates significant difference (p < 0.05; t-test).

Malaria is paralleled by loss of erythrocytes leading to anemia. As shown in Fig. 5, the hematocrit of aurothiomalate-treated mice was significantly reduced. The effect could have been due to enhanced eryptosis or hemolysis. In noninfected erythrocytes aurothiomalate has previously been shown to trigger eryptosis rather than hemolysis [44].

The present study demonstrates that aurothiomalate had only mild effects on the parasite burden and moderately delayed the lethal course of malaria following infection of mice with *P. berghei*. Similar to what has been observed earlier [64], the infection of mice with *P. berghei *was followed by an invariably lethal course of malaria without aurothiomalate treatment. More than 50% of the sodium aurothiomalate-treated animals survived the infection for 30 days, even though they all died until day 44.

The effect of sodium aurothiomalate treatment may in part be due to a toxic effect on the pathogen, which compromises the intraerythrocyte growth of the parasite. As a matter of fact, gold-containing drugs have previously been shown to be toxic for *Plasmodia *[45-57]. Drugs could specifically enter infected erythrocytes, as the pathogen dramatically enhances the permeability of the erythrocyte membrane [1].

Alternatively, sodium aurothiomalate may exert a protective effect by accelerating the death of infected erythrocytes. Phosphatidylserine-exposing erythrocytes are engulfed by macrophages [25,26] and are thus rapidly cleared from circulating blood [27]. As eryptosis mainly affects infected erythrocytes, accelerated eryptosis should decrease the parasitemia and thus favourably influence the course of the disease [29]. On the other hand, eryptosis has been suggested to foster vascular derangements of metabolic syndrome [72].

The discrepancy between the moderate influence of aurothiomalate on parasitemia and the effect on survival of the infected host is suggestive for an additional effect of the drug on mouse survival. Possibly it is in part the anti-inflammatory effect of the drug, which accounts for at least part of the effect on host survival and the stimulation of eryptosis. As a matter of fact, aurothiomalate treatment virtually abolished the increase in TNFα plasma concentration following infection.

## Conclusion

In mice, sodium aurothiomalate delays the lethal course of malaria. Presumably, the effect is not only due to the toxicity for the pathogen and due to stimulation of eryptosis, but may involve the anti-inflammatory activity of the drug.

## List of abbreviations

ANOVA: (analysis of variance); APC: (allophycocyanin); FACS: (fluorescence-activated cell sorter); FL: (fluorescence channel); Hb: (hemoglobin); TNF: (tumor necrosis factor).

## Competing interests

The authors declare that they have no competing interests.

## Authors' contributions

IA carried out the flow cytometry analysis, DB participated in the *in vivo *experiments, SMQ analyzed the TNFα plasma levels, AE maintained the malaria parasite culture. MF and FL conceived the study, participated in its design and coordination and helped to draft the manuscript. All authors read and approved the final manuscript.
